# Increasing the accuracy of the asthma diagnosis using an operational definition for asthma and a machine learning method

**DOI:** 10.1186/s12890-023-02479-4

**Published:** 2023-06-06

**Authors:** Hyonsoo Joo, Daeun Lee, Sang Haak Lee, Young Kyoon Kim, Chin Kook Rhee

**Affiliations:** 1 Division of Pulmonary and Critical Care Medicine, Department of Internal Medicine, College of Medicine, Uijeongbu St. Mary’s Hospital, The Catholic University of Korea, Seoul, Republic of Korea; 2 Departement of Applied Statistics, Yonsei University, Seoul, Republic of Korea; 3 Division of Pulmonary, Critical Care and Sleep Medicine, Department of Internal Medicine, College of Medicine, Eunpyeong St. Mary’s Hospital, The Catholic University of Korea, Seoul, Republic of Korea; 4 Division of Pulmonary and Critical Care Medicine, Department of Internal Medicine, College of Medicine, Seoul St. Mary’s Hospital, The Catholic University of Korea, 222 Banpodaero, Seochogu, Seoul, 06591 Republic of Korea

**Keywords:** Asthma, Conventional operational definition, Machine learning

## Abstract

**Introduction:**

Analysis of the National Health Insurance data has been actively carried out for the purpose of academic research and establishing scientific evidences for health care service policy in asthma. However, there has been a limitation for the accuracy of the data extracted through conventional operational definition. In this study, we verified the accuracy of conventional operational definition of asthma, by applying it to a real hospital setting. And by using a machine learning technique, we established an appropriate operational definition that predicts asthma more accurately.

**Methods:**

We extracted asthma patients using the conventional operational definition of asthma at Seoul St. Mary’s hospital and St. Paul’s hospital at the Catholic University of Korea between January 2017 and January 2018. Among these extracted patients of asthma, 10% of patients were randomly sampled. We verified the accuracy of the conventional operational definition for asthma by matching actual diagnosis through medical chart review. And then we operated machine learning approaches to predict asthma more accurately.

**Results:**

A total of 4,235 patients with asthma were identified using a conventional asthma definition during the study period. Of these, 353 patients were collected. The patients of asthma were 56% of study population, 44% of patients were not asthma. The use of machine learning techniques improved the overall accuracy. The XGBoost prediction model for asthma diagnosis showed an accuracy of 87.1%, an AUC of 93.0%, sensitivity of 82.5%, and specificity of 97.9%. Major explanatory variable were ICS/LABA,LAMA and LTRA for proper diagnosis of asthma.

**Conclusions:**

The conventional operational definition of asthma has limitation to extract true asthma patients in real world. Therefore, it is necessary to establish an accurate standardized operational definition of asthma. In this study, machine learning approach could be a good option for building a relevant operational definition in research using claims data.

## Introduction

Claims data-based studies have become common in health care research during the past decade [1]. Claims data are attractive to researchers because they offer numerous advantages. Claims data have health information that is anonymous, abundant, inexpensive, and widely available in an electronic format [1], and they reflect real-world medical practice. Therefore, these data have been utilized for academic research, post-market surveys, and economic evaluations. However, claims data have several disadvantages; they are not designed for medical research, as they are occasionally missing critical values, and they are under or over-reported in real clinical data [2]. In addition, disease codes in the claims data may not represent a patient’s true disease status. Therefore, building an accurate operational definition of claims data is very important to make these studies more relevant.

Several studies have used national claims data in asthma research [3–7]. In most of these studies, the researchers extracted the asthma patients using a conventional operational definition, which contains two major components. First, they must have the International Classification of Diseases Tenth Revision (ICD-10) codes of asthma as the major diagnosis. Second, they must have been prescribed asthma-related medication. However, there are concerns in asthma research as to whether asthma patients extracted through a conventional operational definition, reflect the reality of all clinical situations.

In this study, we verified the accuracy of the conventional operational definition of asthma by applying it to a real hospital setting. We established an appropriate operational definition that predicts asthma more accurately, using a machine learning technique.

## Methods

### Study design and population

We extracted asthma patients using the conventional operational definition of asthma at Seoul St. Mary’s Hospital (1,356-bed tertiary referral hospital) and St. Paul’s Hospital (301 beds) at the Catholic University of Korea between January 2017 and January 2018. The conventional operational definition of asthma was: 1) ICD-10 codes for asthma; 2) use of more than one drug as an asthma-related medication on at least two outpatient clinic visits [inhaled corticosteroids (ICSs), long-acting β2-agonists (LABAs), ICSs plus long-acting β2-agonists (ICS/LABAs), long-acting muscarinic antagonists (LAMAs), short-acting β2-agonists, short-acting muscarinic antagonists, leukotriene receptor antagonists, systemic β 2-agonists, systemic corticosteroids, or xanthine derivatives] [8].

About 10% of these extracted asthma patients were randomly sampled. We excluded patients < 19-years and patients who did not visit the pulmonology or allergy clinic during the study. We verified the accuracy of the conventional operational definition for asthma by matching the actual diagnosis in a medical chart review. Then, we operated a machine learning approach to predict asthma more accurately. All methods were performed in accordance with the relevant guidelines and regulations. The present study was approved by the Institutional Review Board of Seoul St. Mary’s Hospital and was exempted from informed consent requirements owing to its retrospective design (KC18ZNSI0850).

### Predictors

The predictors for model development were chosen from the results of pulmonary function tests and asthma-related medications in the conventional operational definition of asthma.

### Statistical analysis

We developed a reference model and five machine learning models to predict asthma in the training set (randomly selected 75% of the sample). We fit a logistic regression model for the reference model, including all of the predictors. The predictive models were built with: (1) a decision tree, (2) random forest, (3) extreme gradient boosting (XGBoost), (4) light gradient boosting machine (LGBM), and (5) the CatBoost algorithm. Hyperparameter optimization was performed by a grid search and Bayesian optimization.

A decision tree is a non-parametric supervised learning method used for classification and regression. It is a flowchart-like tree structure, where the internal nodes denote a test of an attribute; each branch represents an outcome of the test, and each leaf node holds a class label. Random forest is an ensemble of decision trees created using bootstrap samples of the training data and random feature selection for inducing the tree. The LGBM was designed to be accurate, efficient, and quick, which are advantages when handling large-scale data. XGBoost is an implementation of a gradient boosting machine and one of the best-performing algorithms utilized for supervised learning. It can be used for both regression and classification problems. CatBoost provides a gradient boosting framework that attempts to solve for categorical features using a permutation-driven alternative compared to the classical algorithm.

We measured the predictive performance of each model by computing the area under the receiver operating characteristic curve (AUC), as well as the accuracy, sensitivity, and specificity of the F1-measure to assess model quality in the test set (remaining 25% of the sample). In addition, to gain stable predictions, we measured the predictive performance of each model with tenfold cross-validation. To test the difference in the evaluation index of each model, this study used bootstrapping on the extra-validation data, prepared 1,000 different test sets in 50 unit sizes or 50,000 bootstrap samples, and applied analysis of variance to test the difference in the average value of the index. Tukey’s post-hoc test was used. All analyses were performed with R version 3.4 software (The R Foundation for Statistical Computing, Vienna, Austria).

## Results

### Baseline characteristics of the study population

**Fig. 1:**
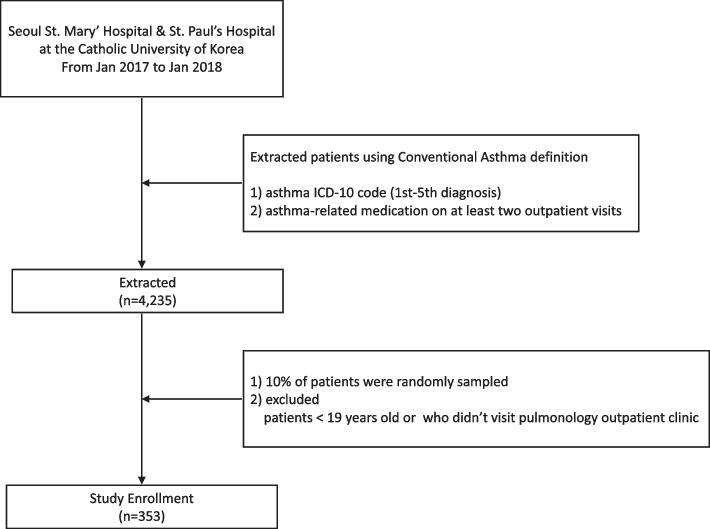
Flow diagram for patient enrollment

A total of 4,235 patients with asthma were identified using the conventional asthma definition. Of these, 353 patients were enrolled (Fig. 1). The baseline characteristics of the enrolled patients are described in Table 1. Among the 353 patients, 49.3% were female and the mean age was 64.6 years; 45.3% were never smokers, 34.8% were current or ex-smokers, and the average smoking pack-years was 18.3. The mean post-bronchodilator (BD) forced expiratory volume in 1 s (FEV1) was 2.01 L (76.3% of predicted) and the mean post-BD FEV1/forced vital capacity was 65.6. Table 2 shows the maintenance drugs of the study population. An ICS/LABA combined inhaler was the most commonly prescribed medication, followed by leukotriene receptor antagonist (LTRA) and LAMA inhalers.

**Table 1:** Baseline characteristics of the study population

Variables	Mean ± SD N (%)
Age, years	64.6 ± 15.2
Female, n (%)	174 (49.3%)
BMI, kg/m^2^	24.1 ± 4.2
Smoking History	
Current smoker	17 (4.8%)
Ex-smoker	106 (30.0%)
Never smoker	160 (45.3%)
Smoking pack-years	18.3 ± 22.5
Pulmonary function	
Post-BD FVC, L	3.08 ± 0.99
Post-BD FVC, % predicted	85.7 ± 18.3
Post-BD FEV1, L	2.01 ± 0.83
Post-BD FEV1, % predicted	76.3 ± 24.5
Post-BD FEV1/FVC, %	65.6 ± 15.7

*BD* Bronchodilator, *BMI* Body mass index, *SD* Standard deviation, *FVC* Forced vital capacity, *FEV1* Forced expiratory volume in 1 s

**Table 2:** Maintenance drugs taken by the study population

Variables	N (%)
ICS/LABA	235 (66.6%)
ICS	30 (8.5%)
LABA	5 (1.4%)
LAMA	83 (23.5%)
LABA/LAMA	26 (7.4%)
SAMA	8 (2.3%)
SABA	58 (16.4%)
Xanthine derivatives	81 (22.9%)
LTRA	184 (52.1%)
Systemic beta agonist	17 (4.8%)
Systemic corticosteroid	27 (7.6%)

*ICS* Inhaled corticosteroid, *LABA* Long-acting beta2-agonist, *LAMA* Long-acting muscarinic antagonist, *LTRA* Leukotriene receptor antagonist, *SABA* Short-acting beta2-agonist, *SAMA* Short-acting muscarinic antagonist

### Actual diagnosis by medical chart review

**Fig. 2:**
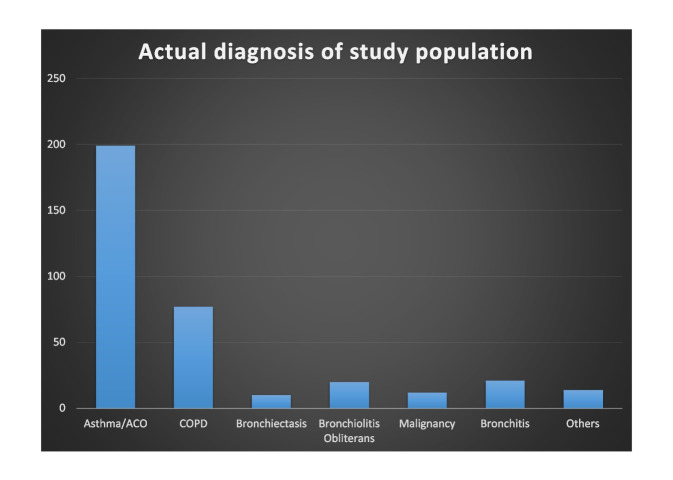
Actual diagnoses of the study population

**Fig. 3:**
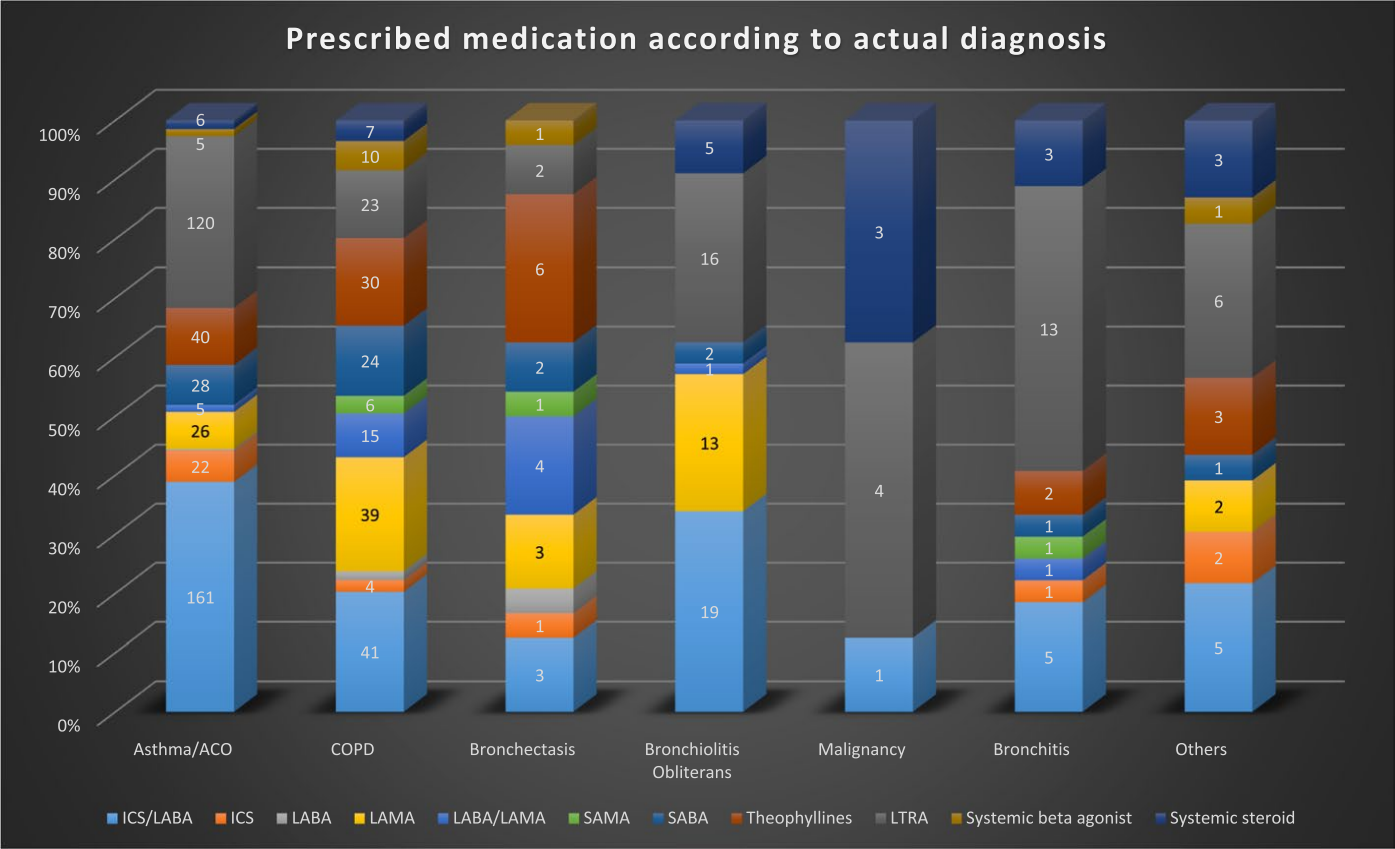
Prescribed medications according to the actual diagnosis

Figure 2 shows the actual diagnoses of the study population. The patients with asthma comprised 56% of the study population, and 44% of the patients did not have asthma. Of these, chronic obstructive pulmonary disease (COPD) was the most common, followed by bronchitis, bronchiolitis obliterans (BO), and other diseases. Additionally, we prescribed asthma medications according to the actual diagnosis (Fig. 3). A LAMA inhaler was the most frequently prescribed medication for COPD and BO patients.

### Predicting asthma patients using a machine learning technique

**Fig. 4:**
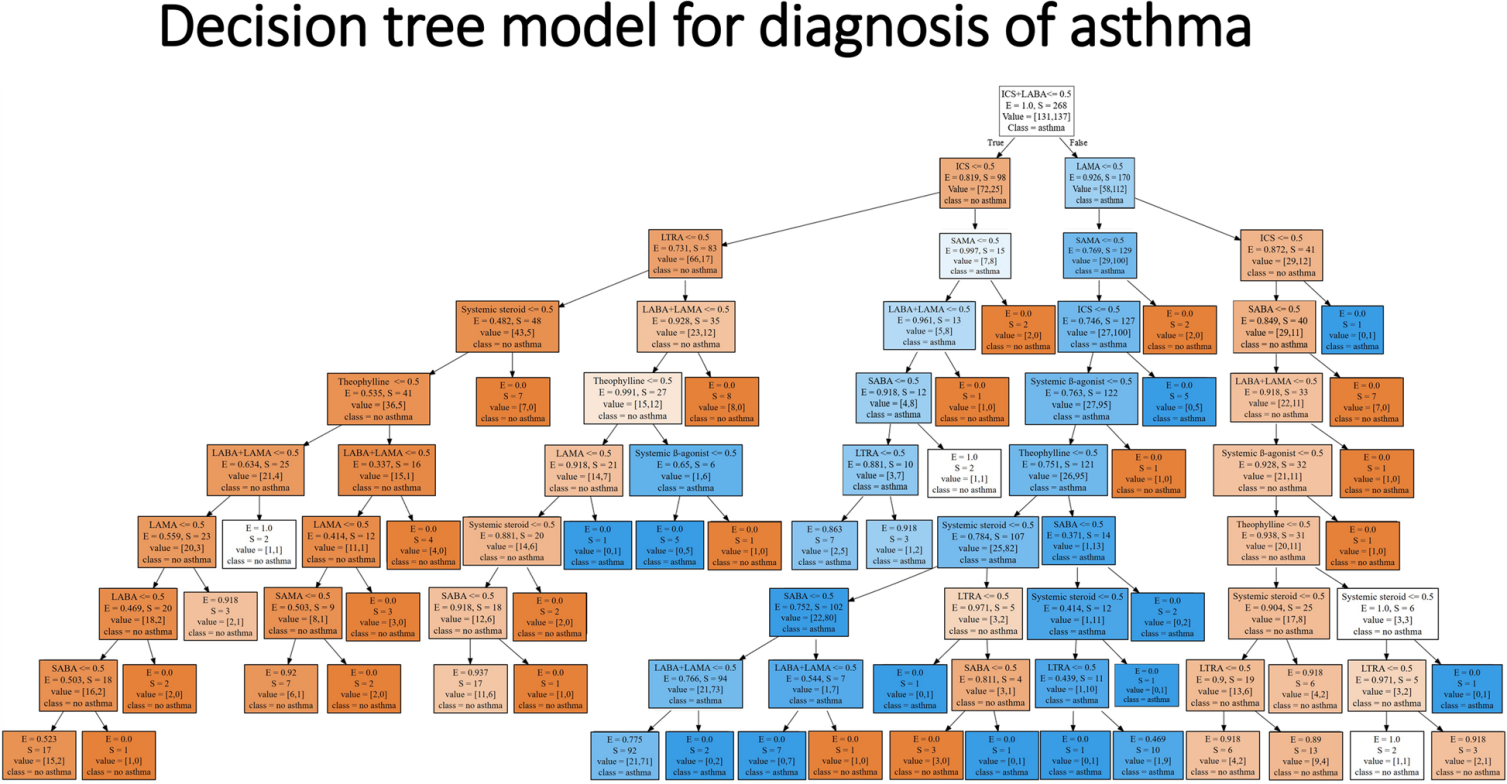
Decision tree model for the asthma diagnosis. Entropy is a measure of the randomness in the information being processed. The higher the entropy, the harder it is to conclude. The sample is the number of data corresponding to the current node. The value list indicates how many samples from a given node fall into each category. Each element in the classification domain is called a class

Two cross-validation methods were used. The predictive abilities of the machine learning models in the asthma diagnosis are summarized in Tables 3 and 4. The LGBM prediction model (grid search) for the asthma diagnosis had an accuracy of 85.9%, an AUC of 91.7%, sensitivity of 79.0%, and specificity of 100%. These results were similar to those of logistic regression and better than those of random forest. Figure 4 shows the decision tree model results (after a grid search) for the asthma diagnosis. The accuracy of the decision tree model was 81.2%. Accuracy was higher than actual accuracy, although accuracy was lower than the other models. The XGBoost predictive model (Bayesian hyperparameter tuning) for the asthma diagnosis showed an accuracy of 87.1%, an AUC of 93.0%, sensitivity of 82.5%, and specificity of 97.9%. This result was better than that of logistic regression. The other models showed better results than actual accuracy. The machine learning models provided higher predictive ability than the conventional operational definition of asthma.

**Table 3:** Predictive abilities of the machine learning models for the asthma diagnosis after a grid search with cross-validation

Model	Accuracy	AUC	Sensitivity (Recall)	Specificity (Precision)
Tree	0.8118	0.8665	0.7193	1.0000
Random forest	0.8353	0.9060	0.7544	1.0000
XGBoost	0.8235	0.8935	0.7368	1.0000
LGBM	0.8588	0.9173	0.7895	1.0000
CatBoost	0.8235	0.9098	0.7368	1.0000

*LGBM* Light gradient boosting model, *XGBoost* Extreme gradient boosting

**Table 4:** Predictive abilities of the machine learning models for the asthma diagnosis after cross-validation with Bayesian hyperparameter tuning

Model	Accuracy	AUC	Sensitivity (Recall)	Specificity (Precision)
Tree	0.8353	0.8712	0.8070	0.9388
Random forest	0.7412	0.8835	0.6140	1.0000
XGBoost	0.8706	0.9301	0.8246	0.9792
LGBM	0.8118	0.8966	0.7193	1.0000
CatBoost	0.8000	0.9029	0.7544	0.9348

*LGBM* Light gradient boosting model, *XGBoost* Extreme gradient boosting

Accuracy was significantly different as a result of bootstrapping, compared with XBG (Bayes), which showed the highest accuracy, but the difference in the AUC between the three models was not significant. The three models with the highest performance had a similar degree of accuracy (Table 5).

**Table 5:** Predictive abilities of the machine learning models for the asthma diagnosis after bootstrap cross-validation

Model	Accuracy	AUC	Sensitivity (Recall)	Specificity (Precision)	F1 score
XGBoost	0.869421	0.893428	0.823082	0.978909	0.89388
LGBM	0.858168	0.894258	0.788516	1.0000	0.881333
(Logi)	0.858168	0.894258	0.788516	1.0000	0.881333
Tree	0.834608	0.849462	0.80581	0.938875	0.86682
Random forest	0.822236	0.867463	0.734925	1.0000	0.846677

*LGBM* Light gradient boosting model, *XGBoost* Extreme gradient boosting

### Important predictive variables

**Fig. 5:**
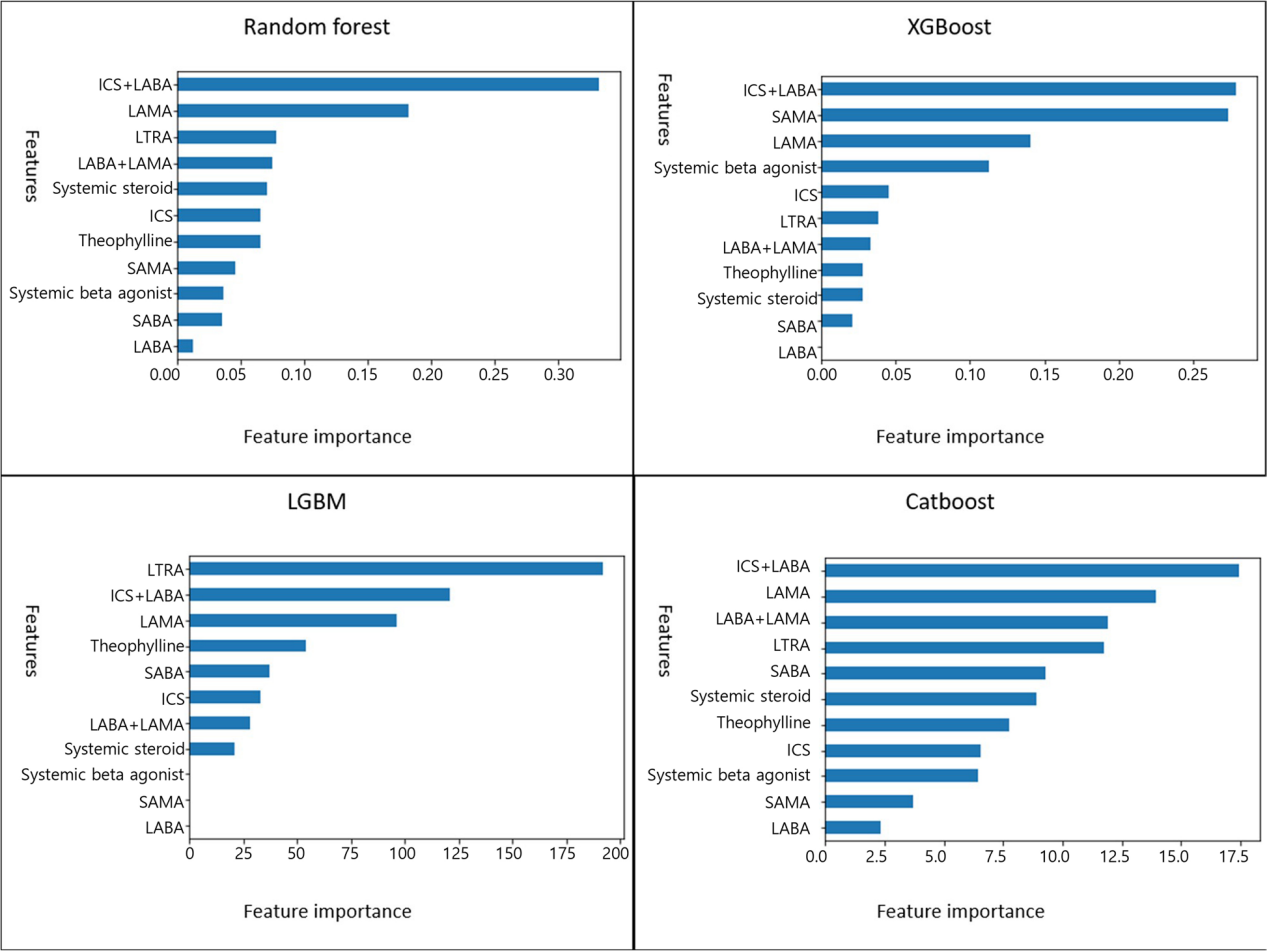
Variable importance based on the machine learning models for the asthma diagnosis after a grid search with cross-validation

**Fig. 6:**
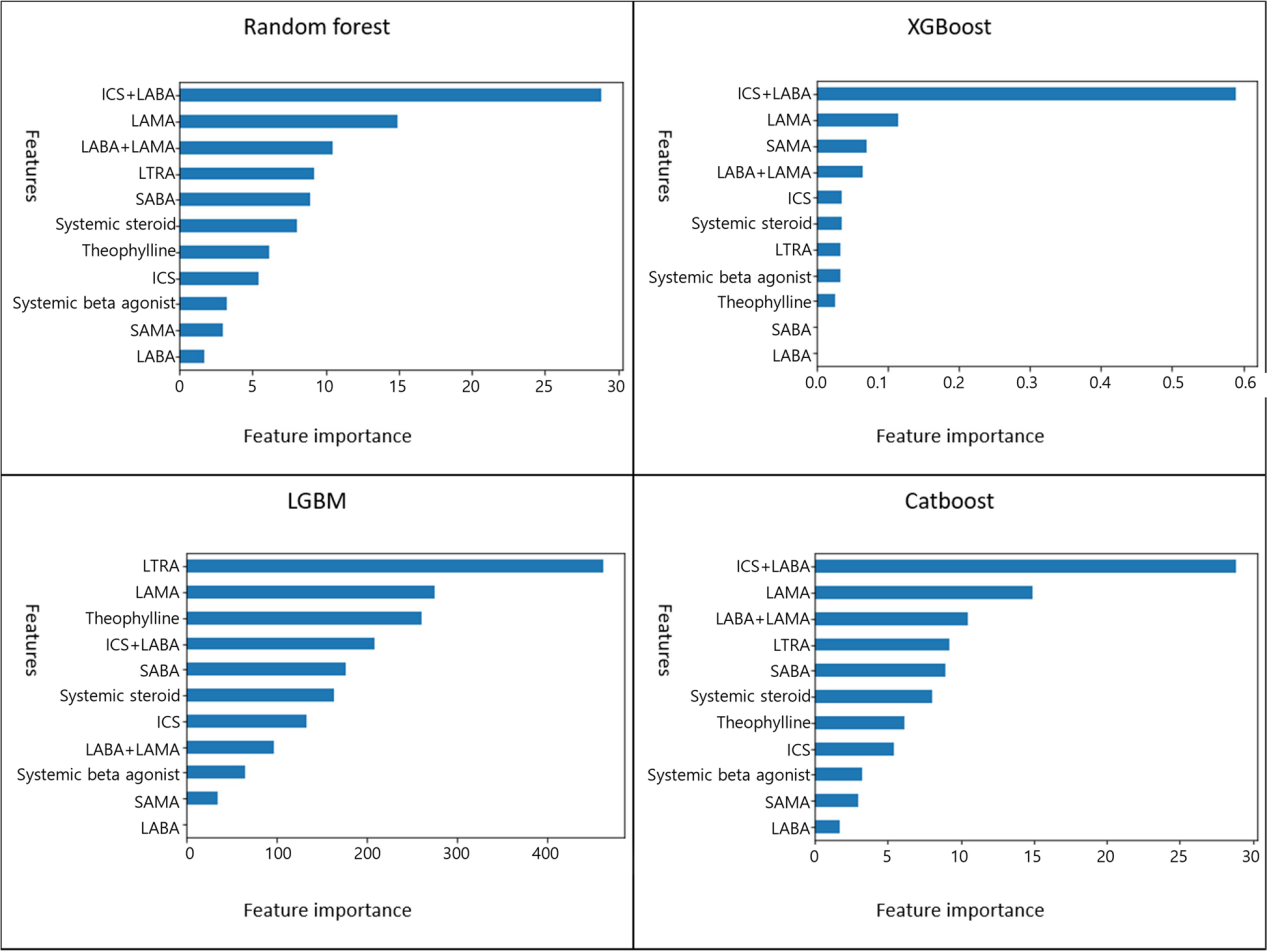
Variable importance based on the machine learning models for the asthma diagnosis after cross-validation with Bayesian hyperparameter tuning

Figures 5 and 6 indicate the important variables for predicting the asthma diagnosis. The major explanatory variables were ICS/LABA, LAMA, and LTRA for a proper diagnosis of asthma. The most significant variable for a proper diagnosis of asthma in the logistic regression model was using an ICS/LABA inhaler, followed by an ICS inhaler (Table 6). This result is similar to the result of the machine learning technique.

**Table 6:** Variable importance using logistic regression to predict the asthma diagnosis more accurately

	Coefficient	*P*-value
(Intercept)	− 0.56	0.32552
PFT	− 0.0538	0.91038
ICSLABA	1.4263	0.00007
ICS	1.3623	0.02046
LABA	− .05754	0.70679
LAMA	− 1.463	0.00005
LABALAMA	− 1.0956	0.6184
SAMA	− 1.4257	0.18140
SABA	− 0.2296	0.60339
Theophylline	0.3295	0.38583
LTRA	0.5134	0.07581
Systemic beta agonist	− 0.5349	0.44372
Systemic steroid	− 0.9765	0.08474

*ICS* Inhaled corticosteroid, *LABA* Long-acting beta2-agonist, *LAMA* Long-acting muscarinic antagonist, *LTRA* Leukotriene receptor antagonist, *SABA* Short-acting beta2-agonist, *SAMA* Short-acting muscarinic antagonist

## Discussion

Claims data can be used to examine the current status and trends that reflect the actual health care environment rather than a limited experimental environment. However, the accuracy of the disease diagnosis included in claims data remains controversial. An operational definition can be used to confirm the presence or absence of disease. However, this approach has some limitations. The number of extracted patients decreases, which dilutes the advantage of claims data as big data when placing conditions for extracting patients using the operational definition. Although extracting with simple conditions leads to extracting more patients, it results in including more unintended patients.

In this study, we examined the actual proportion of patients by comparing the patients sampled based on an operational definition and the data in their medical charts. The data were analyzed based on an operational definition frequently used in previous research. Patients with asthma accounted for only 56% of the cases. In other words, 44% were erroneously included as asthma patients even if they did not have asthma. To overcome this problem, we used a machine learning approach that resulted in an observable increase in accuracy. To the best of our knowledge, this is the first study to compare claims data to those in actual medical charts. Additionally, this is the first study to utilize a machine learning method to increase the accuracy of the diagnosis of patients sampled via an operational definition.

The key predictor in the machine learning models was the ICS/LABA inhaler. ICS/LABA are basic drugs for treating asthma. Therefore, many asthmatic patients use ICS/LABA. ICS/LABA are often used for COPD, and ICS/LABA are still in use for some airway diseases. Thus, determining whether someone has asthma with ICS/LABA is less accurate. Another key predictor was LAMA. The use of LAMA was identified by logistic regression as least associated with the diagnosis of asthma. LAMA is the most basic treatment for COPD but is used as an add-on in severe asthma cases. Previous studies that used claims data were inconsistent in including LAMA as an asthma-related drug. Nevertheless, the sample sizes of two other studies conducted during the same time were different due to a difference in the operational definition. The proportion of LAMA use is 20% in tertiary hospitals [7]. LAMA use has been excluded in studies that did not include LAMA [3]. In our study, a review of the medical charts of the patients in the sample showed that COPD was the second most common wrong entry after asthma and that some COPD patients used LAMA. However, if LAMA was removed, 13.1% (26/199) of actual asthma patients would be excluded. In particular, severe asthma patients would be excluded. However, if a machine learning method is used, the accuracy of the asthma diagnosis increases, even when patients are sampled via an operational definition that included LAMA.

According to our study, the accuracy of previous definition of asthma by claim data was only 56%. Thus, many previous studies by conventional definition may not represent real asthma patients. This is one of the reasons why we performed this study. By applying this new method, researchers can analyze more accurate characteristics of asthma patients in the future.

Our study had several limitations. First, the patients were older. The prevalence of asthma increases with age; however, the patients in this study were much older than those included in previous studies that used claims data [9, 10]. The higher the age of an asthma patient, the more difficult it is to differentiate asthma from COPD [11–14]. However, even when considering these factors, accuracy increased in our study. Second, only the patients in a referral hospital were extracted and analyzed. There are differences in the use of medications in primary, secondary, and tertiary hospitals [7]. The frequency of use of LAMA in a primary hospital is remarkably low. The key predictor in the machine learning methods was LAMA. Thus, the effectiveness of machine learning may seem exaggerated, but the increase in diagnostic accuracy is undeniable. Third, our study used the multiple machine learning models and hyper-parameter optimization techniques may increase the risk of overfitting the models to the training data, which may result in poorer performance when applied to new data. We will keep this in mind when applying these techniques to other studies. Fourth, the sample size was small. Because it took a long time to review the medical charts of all patients in the sample, only 10% of the medical charts were reviewed randomly. However, this sample was sufficiently large to apply machine learning.

## Conclusion

The conventional operational definition of asthma has a limited range, so that true asthma patients in the real world may be extracted. Therefore, it was necessary to establish an accurate standardized operational definition of asthma. In this study, a machine learning approach was a good option for building a relevant operational definition using claims data.

## Data Availability

The datasets used and/or analyzed during the current study are available from the corresponding author on reasonable request.

## Abbreviations

AUC: Area under the receiver operating characteristic curve
BD: Bronchodilator
BMI: Body mass index
BO: Bronchiolitis obliterans
COPD: Chronic obstructive pulmonary disease
FEV: Forced expiratory volume in 1 s
FVC: Forced vital capacity
ICD-10: International Classification of Diseases Tenth Revision
ICS: Inhaled corticosteroid
LABA: Long-acting β2-agonist
LAMA: Long-acting muscarinic antagonist
LGBM: Light gradient boosting machine
LTRA: Leukotriene receptor antagonist
SABA: Short-acting β2-agonist
SAMA: Short-acting muscarinic antagonist

## References

[1] Ferver K, Burton B, Jesilow P (2009). The use of claims data in healthcare research. Open Public Health J.

[2] Lee J, Lee JS, Park SH, Shin SA, Kim K (2017). Cohort profile: the National Health Insurance Service-National Sample Cohort (NHIS-NSC), South Korea. Int J Epidemiol.

[3] Choi JY, Yoon HK, Lee JH, Yoo KH, Kim BY, Bae HW, Kim YK, Rhee CK (2017). Current status of asthma care in South Korea: nationwide the health insurance review and assessment service database. J Thorac Dis.

[4] Choi JY, Yoon HK, Lee JH, Yoo KH, Kim BY, Bae HW, Kim YK, Rhee CK (2018). Nationwide pulmonary function test rates in South Korean asthma patients. J Thorac Dis.

[5] Choi JY, Yoon HK, Lee JH, Yoo KH, Kim BY, Bae HW, Kim YK, Rhee CK (2018). Nationwide use of inhaled corticosteroids by South Korean asthma patients: an examination of the health insurance review and service database. J Thorac Dis.

[6] Park HJ, Byun MK, Kim HJ, Ahn CM, Rhee CK, Kim K, Kim BY, Bae HW, Yoo KH (2018). Regular follow-up visits reduce the risk for asthma exacerbation requiring admission in Korean adults with asthma. Allergy Asthma Clin Immunol.

[7] Cho EY, Oh KJ, Rhee CK, Yoo KH, Kim BY, Bae HW, Lee BJ, Choi DC, Lee H, Park HY (2018). Comparison of clinical characteristics and management of asthma by types of health care in South Korea. J Thorac Dis.

[8] Rhee CK, Yoon HK, Yoo KH, Kim YS, Lee SW, Park YB, Lee JH, Kim Y, Kim K, Kim J (2014). Medical utilization and cost in patients with overlap syndrome of chronic obstructive pulmonary disease and asthma. COPD.

[9] Kim S, Kim J, Kim K, Kim Y, Park Y, Baek S, Park SY, Yoon SY, Kwon HS, Cho YS (2013). Healthcare use and prescription patterns associated with adult asthma in Korea: analysis of the NHI claims database. Allergy.

[10] Lee E, Kim A, Ye YM, Choi SE, Park HS (2020). Increasing prevalence and mortality of asthma with age in Korea, 2002–2015: a nationwide, population-based study. Allergy Asthma Immunol Res.

[11] Gillman A, Douglass JA (2012). Asthma in the elderly. Asia Pac Allergy.

[12] Akgun KM, Crothers K, Pisani M (2012). Epidemiology and management of common pulmonary diseases in older persons. J Gerontol A Biol Sci Med Sci.

[13] Oraka E, Kim HJ, King ME, Callahan DB (2012). Asthma prevalence among US elderly by age groups: age still matters. J Asthma.

[14] Weiner P, Magadle R, Waizman J, Weiner M, Rabner M, Zamir D (1998). Characteristics of asthma in the elderly. Eur Respir J.

